# Persistent DNA Damage after High Dose *In Vivo* Gamma Exposure of Minipig Skin

**DOI:** 10.1371/journal.pone.0039521

**Published:** 2012-06-27

**Authors:** Emad A. Ahmed, Diane Agay, Gerrit Schrock, Michel Drouet, Viktor Meineke, Harry Scherthan

**Affiliations:** 1 Institut für Radiobiologie der Bundeswehr in Verbindung mit der Universität Ulm, München, Germany; 2 IRBA-antenne La Tronche - CRSSA, La Tronche, France; University Medical Center Hamburg-Eppendorf, Germany

## Abstract

**Background:**

Exposure to high doses of ionizing radiation (IR) can lead to localized radiation injury of the skin and exposed cells suffer dsDNA breaks that may elicit cell death or stochastic changes. Little is known about the DNA damage response after high-dose exposure of the skin. Here, we investigate the cellular and DNA damage response in acutely irradiated minipig skin.

**Methods and Findings:**

IR-induced DNA damage, repair and cellular survival were studied in 15 cm^2^ of minipig skin exposed *in vivo* to ∼50 Co-60 γ rays. Skin biopsies of control and 4 h up to 96 days post exposure were investigated for radiation-induced foci (RIF) formation using γ-H2AX, 53BP1, and active ATM-p immunofluorescence. High-dose IR induced massive γ-H2AX phosphorylation and high 53BP1 RIF numbers 4 h, 20 h after IR. As time progressed RIF numbers dropped to a low of <1% of keratinocytes at 28–70 days. The latter contained large RIFs that included ATM-p, indicating the accumulation of complex DNA damage. At 96 days most of the cells with RIFs had disappeared. The frequency of active-caspase-3-positive apoptotic cells was 17-fold increased 3 days after IR and remained >3-fold elevated at all subsequent time points. Replicating basal cells (Ki67+) were reduced 3 days post IR followed by increased proliferation and recovery of epidermal cellularity after 28 days.

**Conclusions:**

Acute high dose irradiation of minipig epidermis impaired stem cell replication and induced elevated apoptosis from 3 days onward. DNA repair cleared the high numbers of DBSs in skin cells, while RIFs that persisted in <1% cells marked complex and potentially lethal DNA damage up to several weeks after exposure. An elevated frequency of keratinocytes with persistent RIFs may thus serve as indicator of previous acute radiation exposure, which may be useful in the follow up of nuclear or radiological accident scenarios.

## Introduction

High dose radiation exposures known from accidents and radiotherapy have highlighted the cutaneous radiation reaction being of importance for the clinical prognosis and sometimes for the survival of IR-exposed accident victims [1]. After exposure to ionizing radiation (IR), a subsequent dose-dependent cutaneous radiation reaction includes early effects such as inflammation and erythema and stochastic long-term effects such as fibrosis, keratosis and skin cancer [2]. In addition to routine therapeutic radiation exposures, individuals may also experience high-dose exposures in accident scenarios leading to localized radiation wounds [3]–[5]. The investigation of treatment options for the cutaneous radiation syndrome requires animal models, of which pig skin is the nearest match to human skin in structure and radiation response [6], [7].

Acute exposure of English Large White pig skin to 17–27 Gy X-rays has been shown to produce cutaneous radiation syndrome that involves moist desquamation after 17 days, healing at 32 days, and reappearance of moist desquamation between 42 and 70 days [8], [9]. The associated histological changes of epidermal cell morphology, linear density, and mitotic activity separate into degenerative (cell loss), regenerative (cell replacement), and post-regenerative (hyperplasia followed by cell loss) phases [6]. Strain-related differences for the incidence of moist desquamation are known from the English Large White and the Göttingen minipig strains. While for the former the ED50 values for moist desquamation are about 27 Gy, the Göttingen minipig displays ED50 values of about 39 Gy [8]. Recently, a minipig model for human radiation accident scenarios was established and allows testing treatment options of radiation burns [10]. In this model approx. 50 Gy of acute Co-60 γ-exposure is used to induce moist skin desquamation 10 weeks after IR of Göttingen minipigs [10], [11].

Although the cutaneous radiation response after radiotherapy has been extensively studied [12]–[14], little information is available about the *in vivo* DNA damage response after acute high-dose exposure of the skin of large animal models [15]. DNA damage is an important ionizing radiation-mediated lesion directing cellular survival and stochastic effects, especially through formation of double stranded DNA breaks (DSBs). Genotoxic exposures lead to DSB formation which elicits a DNA damage response (DDR) that persists up to completion of repair or cell death (reviewed by [16], [17]). Misrepair of DSBs can be mutagenic and may thus lay the ground for cancer formation years later. Immediately after DSB induction, histone H2AX is phosphorylated at serine 139 (now termed γ-H2AX; [18]) by the ATM kinase, leading to γ-H2AX formation in the chromatin surrounding DSBs. These chromatin regions can be visualized as discrete nuclear foci by immunofluorescence microscopy [12], [19], with each γ-H2AX focus representing at least one DSB in low dose IR scenarios [20], [21], while at higher doses more than one DSB will be contained in a γ-H2AX focus [22]. After a DSB has been repaired, γ-H2AX molecules are dephosphorylated or turned over, leading to the disappearance of radiation-induced foci [23]–[25].

A subclass of radiation-induced foci (RIF) may persist for hours or even days and are likely the consequence of complex DNA damage that is difficult to repair, especially after high-dose exposures [26], [27]. IR-induced γ-H2AX or 53BP1 RIF ≥24 hours after IR are thus considered as indicators for delayed or impaired DSB repair due to complex DNA damage [12], [28]–[30] which may be lethal to the cell [31]. γ-H2AX RIF in irradiated mouse skin have been noted up to 7 days post exposure and it has been proposed that they may be used as a biodosimeter in accident scenarios [30]. Similar observations have been made for the 53BP1 DNA damage sensor protein [14], [32], [33] that also forms foci around DSBs to instigate the DNA damage response [34], [35].

While the knowledge about the induction, regulation and function of γ-H2AX foci is constantly growing in *in vitro* and in vivo model systems (e.g., [15], [36]–[38]), few data are available from high-dose exposed human or large animal model skin. In this report, we investigate *in vivo* DNA damage induction and repair in the epidermis of the Göttingen minipig after localized exposure to 50 and 53 Gy of Co-60 γ irradiation [10]. Our data extend previous observations made in the mouse model [30] and show that acute high-dose irradiation induces complex DNA damage that may persist up to months in a subset of terminally differentiating corneocytes of large animal skin.

## Results

### RIF Turnover after Acute Gamma Ray Exposure of the Skin

To investigate the *in vivo* DNA damage response elicited by an acute 50 Gy Co-60 gamma IR dose to the skin, we studied DNA damage and repair-associated radiation-induced focus formation in skin biopsies from a radiation wound treatment study in the Göttingen minipig [10]. The dose delivered to the surface of the 3.5×5 cm investigated skin region was 50 Gy in 2 pigs and 53 Gy in a third pig, and biopsies were available from time points 4 h, 3 days, 28, 49, 70 and 98 days post irradiation. Additionally, we investigated two *ex vivo* 50 Gy irradiated skin sample 20 h post IR, since the DNA damage response and fast repair in skin and other tissues is usually encompassing 1–3 days (e.g., [30], [38]).

**Figure 1:**
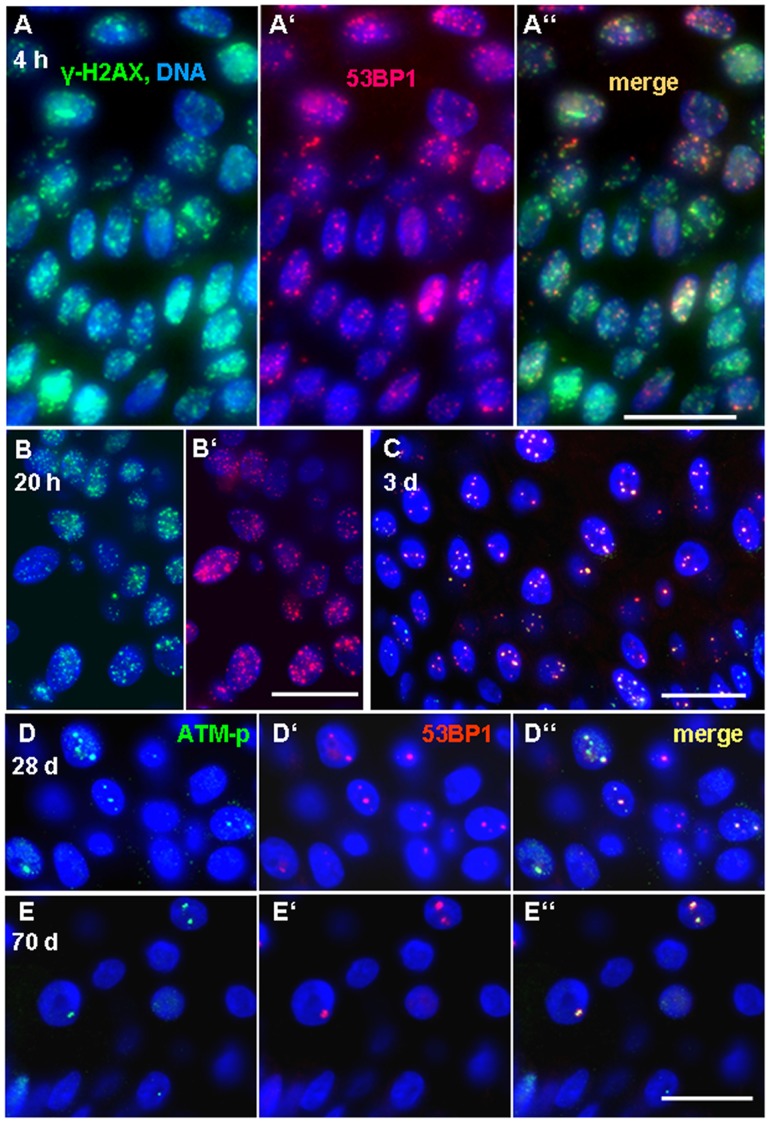
Evolution of DNA damage-dependent γ-H2AX (green) and 53BP1 (red) focus formation as analyzed by immunofluorescence and 3D maximum image projection. (A-A’’) Skin section displaying strong patchy γ-H2AX and spotty 53BP1 fluorescence 4 h after 50 Gy irradiation. Separated green (A) and red (A’) channels, as well as RGB merge (A’’) are shown. Numerous nuclei contain large γ-H2AX patches and diffuse labeling, but a fraction of nuclei reveals countable foci. (B) Numerous distinct γ-H2AX and 53BP1 RIF/nucleus are seen in a skin sample 20 h after *ex vivo* irradiation with 50 Gy in the corresponding green and read (B’) channel images. (C) Skin biopsy 3 days after in vivo IR displaying a lower number of RIF/nucleus indicative of progression of DNA repair. RGB image, the yellowish color at RIFs reflects γ-H2AX and 53BP1 colocalization. (D-E) Damage persistence and ongoing DDR as revealed by active ATM-p (green) and 53BP1 (red) colocalization at large RIFs of keratinocytes 28 days (D-D’’) and 70 days (E-E’’) post IR. Nuclear DNA is labeled in blue (DAPI). Magnification bar: 20 µm.

**Figure 2:**
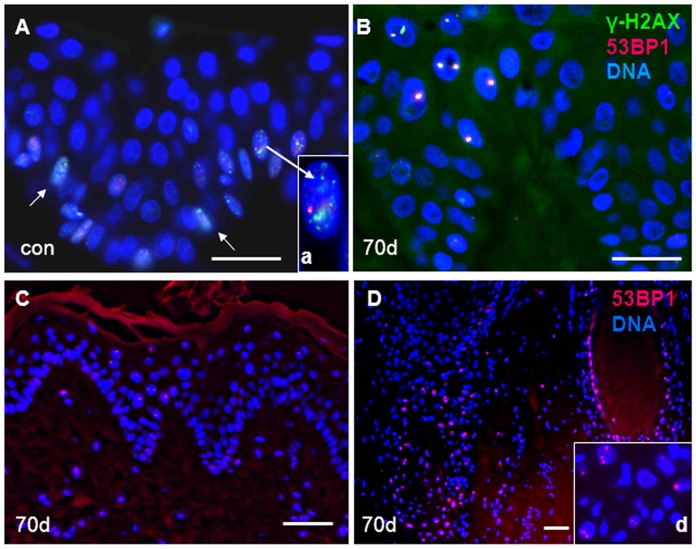
(A) Non-irradiated control skin (con) showing nuclei (blue) with numerous small γ-H2AX (green) and fewer 53BP1 (red) foci in cells at the basal layer of the epidermis (arrows) indicating γ-H2AX formation due to collapsed replication forks in S-phase cells. Such replicating nuclei (compare Fig. 4) with numerous small γ-H2AX foci (inset, arrow) were excluded from the enumerations of all time points. (B-D) Skin sections 70 days after IR. (B) Keratinocyte nuclei of the epidermis displaying one or two large RIFs with γ-H2AX and 53BP1 colocalization due to complex DNA damage in nuclei of the basal and spinous layer. (C) Epidermis at lower magnification with 53BP1 RIF carrying nuclei in all layers. (D) Many nuclei of hair sheath cells carry strong and numerous 53BP1 RIF. The inset displays magnified part of the hair bulb compartment. Magnification bar: 20 µm.

DNA damage and repair was studied by γ-H2AX and 53BP1 RIF formation using maximum projection imaging of foci (Fig. 1, 2). Small S-phase-related γ-H2AX foci were seen in a subset of keratinocytes of the stratum basale of the epidermis (Fig. 2A) in control and samples without massive RIF formation from more advanced time points. Ki67 and 53BP1 co-staining of control and irradiated samples (Fig. S1) confirmed the origin of these small foci patterns from S-phase cells (Fig. 2A). Such foci relate to DNA damage at stalled/collapsed replication forks during S-phase (see e.g., [39]), and since these foci patterns are not IR-related they were excluded from all RIF enumerations.

**Figure 3:**
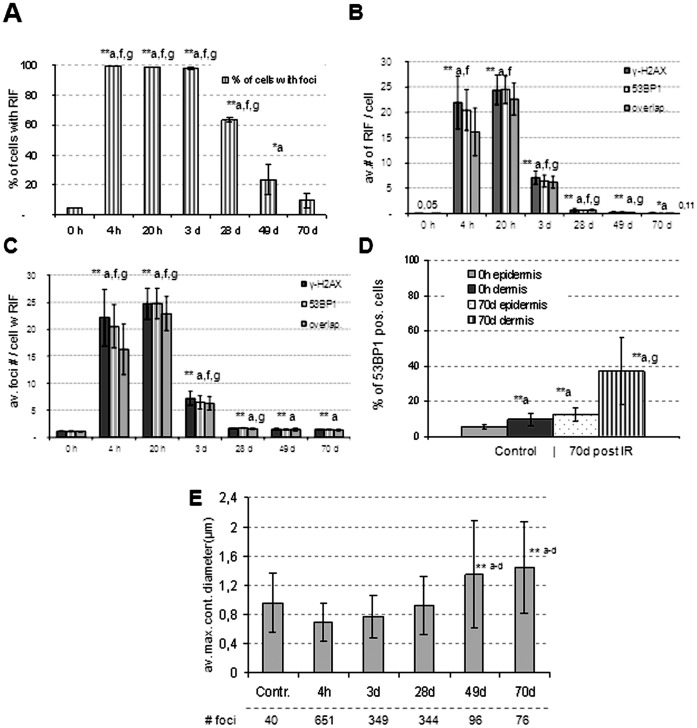
DNA damage response in epidermis sections of 140 µm unit length after in vivo exposure to 50 Gy γ-irradiation studied by radiation-induced focus analysis. The mean and SD of 3 pigs are presented, except for the 20 h and 3 day values (2 pigs). (A) 4 h and 20 h post IR all cells contained RIF. This percentage dropped after 3days post IR. (B) Average number of γ-H2AX and 53BP1 RIF per cell in skin biopsies at the consecutive time points investigated. At 4 h post IR the focus number was derived from cells with distinct γ-H2AX labeling (see text, Fig.1A). “Overlap” addresses foci with γ-H2AX and 53BP1 signal colocalization, which is complete at late time points. (C) Average foci numbers in keratinocytes that carry ≥1 RIF are significantly increased relative to control, 4 h and 20 h time points (**p<0.001). (D) Percentage of cells carrying ≥1 53BP1 RIF at 70 days post IR. There is a significant difference (**, p<0.001) between keratinocytes of the hair sheath (70d dermis) and all other conditions. (E) At ≥49 days post IR the mean 53BP1 foci diameter (maximum contour radius ± SD) is significantly increased (**, p<0.002). Error bars reflect differences of 3 different animals. Significant differences were noted ^a^ relative to control, ^b^ relative to 4 h, ^c^ to 3 days, ^e^ to 28 days, ^f^ to 49 days and, ^g^ relative to 70 days.

**Table 1:** Average number of DSB-related foci/cell in pig epidermis.

time post IR	Con	4 h	20 h	3 d	28 d	49 d	70 d	98 d
Pig # (skin surface dose)				**γ-H2AX**				
P212 (50 Gy)	0.04	17.06	22.44	6.77	0.59	0.26	0.14	0.04
P213 (50 Gy)	0.05	21.45	26.50	7.43	0.51	0.08	0.07	0.05
P201 (53 Gy)	0.06	27.49	–	–	1.02	0.36	0.11	–
				**53BP1**				
P212 (50 Gy)	0.04	17.82	22.57	6.22	0.64	0.26	0.15	0.03
P213 (50 Gy)	0.05	18.48	26.50	6.65	0.63	0.10	0.06	0.04
P201 (53 Gy)	0.03	25.11	–	–	0.66	0.34	0.07	–

−, sample not available. Con, non-irradiated control.

**Figure 4:**
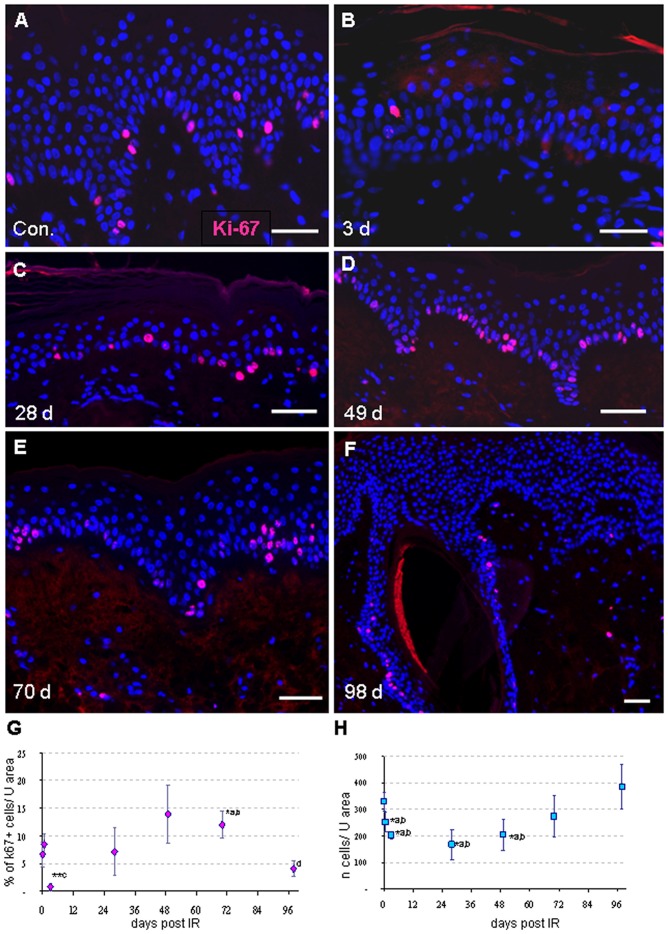
IR-induced changes of proliferation index (Ki67) and in epidermal cellularity as displayed by nuclear counterstaining (DAPI, blue). (A) Control minipig skin samples showing Ki67-positive cells (pink) at the basal layer of the densely populated epidermis. (B) Minipig skin sample 3 days post IR displaying a dearth of replicating cells, while at 28 (C), 49 (D) and 70 (E) days proliferating cells are abundant. (F) At 98 days epidermal hyperplasia was associated with a drop of replicating cell number. Magnification bar for all details: 20 µm. (G) Frequency of Ki67 positive (+) cells per unit area revealing a significant reduction (**p<0.001) of Ki-67-positive cells 3 days after IR relative to control and 4 h. A raise of replicating cells was noted ≥ 49 days post IR being significant at day 70 relative to control and the earlier time points. The fraction of replicating cells was significantly reduced at 96 days post IR, being associated with epithelia hyperplasia. (H) Epidermal cellularity during the time window investigated as determined by enumeration of the total number of DAPI-positive nuclei (blue, A-F) of keratinocytes per 169 µm unit contour length (average of 3 pigs). There is a significant decrease in the cellularity of the epidermis from 3 days up to 28 days post IR, as compared to control. A constant raise of the cellularity of the epidermis was observed from 28 days post irradiation onwards. The mean and SD of 3 pigs is given, except for 3 days (2 pigs); *, p<0.05; **, p<0.001. (G,H) Significant difference: ^a^ relative to control, ^b^ relative to 98 d, ^c^ relative to all other time points, ^d^ relative to 4 h, 3d, 48d and 72 days.

At 4 h, 20 h and 3 days post IR all cells in the skin biopsies displayed formation γ-H2AX and numerous 53BP1 foci (Figs 1A-C, 3A). At 4 h post IR, most cells (54% and 63% in the two 50 Gy-exposed pigs; 73% in the 53 Gy-exposed pig) displayed strong phosphorylation of histone H2AX often leading to a strong patchy γ-H2AX signal that included confluence of numerous foci (Fig. 1 A). This strong kinase-dependent DNA damage response often rendered enumeration of RIF in 3D projection images impossible, and may relate to the high number of DSBs seeded by ∼50 Gy photon irradiation that is expected to induce about 2000 DSBs/nucleus [40]. Nonetheless, about 40% of nuclei displayed distinct RIF patterns that still could be enumerated in 3D maximum projection images obtained from epidermis regions of 140 µm contour length. In average there were about 22 distinct γ-H2AX foci and 20 53BP1 foci (Fig. 1A; Fig.3B), while both DNA damage markers generally showed colocalization (Fig. 3B). When comparing the individual γ-H2AX RIF numbers per cell among the three pigs, the mean γ-H2AX RIF yield per cell was highest in the epidermis exposed to 53 Gy (in average 27.5 RIF), while the two 50Gy-exposed biopsies showed lower γ-H2AX RIF number (17.6 and 21.5 RIF; Table 1). Likewise, the average 53BP1 foci number was highest in the biopsies of the pig exposed to 53 Gy, while the 53BP1 RIF numbers closely matched in the 4 h skin biopsies of the two 50 Gy exposed pigs, (Table 1), rendering a quantitative difference even at these high-dose exposures. Inter-cellular differences in overall γ-H2AX intensities and RIF numbers may relate to individual differences in kinase activity early after exposure, and the different fractions of cells showing enumerable γ-H2AX RIFs in biopsies at 4 h post 50 Gy may relate to the phosphorylation of H2AX not yet being fully blown shortly after such high doses, or that fast DNA repair has already created differences among the individual cellular overall γ-H2AX signals.

Ex vivo 50Gy-irradiation of biopsies of normal skin was performed for two pigs followed by fixation after 20 h of organ culture. RIF number enumeration revealed foci in all cells (Fig. 1B, 3A) with the average γ-H2AX and 53BP1 RIF numbers exceeding those seen at 4 h (Fig. 3B). Nuclei with strong dispersed γ-H2AX formation, as seen at 4 h, were absent in the 20 h *ex vivo* samples (Fig. 1B), suggesting advancement of the DNA damage response. Three days after 50 Gy *in vivo* exposure (2 pigs) we noted a significant recession of the average RIF numbers (Fig. 1C) relative to the 4 h and 20 h time points (Fig. 3B; Table 1) indicating further progress of DNA repair.

At even more advanced time points (28, 49 and 70 days) after IR the average RIF number per epidermal keratinocyte had significantly dropped (Fig. 1D,E; 3C,D), but there remained at > two-fold increase of the average RIF number (Fig. 3B), a significant difference (p<0.001) to control and the early points. At 96 days post irradiation the average RIF numbers had returned to the level of non-irradiated control (Table 1).

At 49 and 70 days post IR there still was a small but significant fraction of keratinocytes that showed γ-H2AX and 53BP1 colocalization at one or two large RIFs per nucleus (Fig. 2 B,C). Again the average RIF number was higher in the 53 Gy-exposed skin relative to the 50 Gy samples (Table 1), reflecting the dose difference.

Furthermore, we noted that the percentage of 53BP1 foci-bearing keratinocytes (≥ one 53BP1 focus/cell) was significantly higher in epidermal hair sheath cells than in epidermal keratinocytes (Fig. 2C,D; Fig. S2), both in non-irradiated samples and samples at 70 days post IR (Fig. 3D). This difference between hair sheath cells and superficial epidermis seems to be independent of dose build up, since it was also evident for hair sheath cells near the skin surface (not shown).

### Large Persistent Foci Reflect Complex DNA Damage

The persistent DNA damage-related foci at late time points exhibited a significantly increased diameter as compared to the earlier time points (Fig. 3E). Interestingly, such large-sized foci were seen in nuclei of keratinocytes from the irradiated area in the 28 day samples and up to 10 weeks post-irradiation (Fig. 3C), but were rarely encountered in non-exposed samples from the same minipig (Fig. 2A). Digital image analysis of maximum projection images revealed a significant increase of the mean maximum contour diameter of foci at 49 and 70 days after IR (p<0.002) relative to the control and the 4, 20 h and 3day time points post IR (Fig. 3E). Since such large persistent foci are likely indicative of complex and potentially lethal DNA damage that contains base lesions, ssDNA and dsDNA breaks as well as abasic sites [31], [41], we investigated whether these foci harbor ongoing DNA damage signaling by costaining of 53BP1 or γ-H2AX with active ATM (phosphorylated Ser 1981; [42]). These experiments revealed the colocalization of active ATM-p with 53BP1 (Fig.1D, E) and with γ-H2AX (Fig. 2B) in large foci, indicating the accumulation of chromatin with complex DNA damage in the one or two RIFs per damage carrying cell and ongoing DNA damage signaling in these chromatin subcompartments up to 10 weeks post IR.

### Demise of Replicating Cells Early after IR Precedes Reduced Keratinocyte Cellularity after High Dose of Irradiation

Since high dose irradiation will reduce the proliferation capacity and survival of keratinocytes, especially of the proliferative epidermal stem cells of the basal layer, we next studied proliferation in control and irradiated skin (Fig. 4). Estimation of Ki-67-positive (S-phase) cells revealed a highly significant reduction of the percentage of Ki67-positive cells per unit area 3 days after IR, as compared to control and the more advanced time points (Fig. 4G). This indicates an impairment of proliferation by high-dose exposure, especially in the mitotically active stem cells. From 28 days up to 70 days after IR, there was a constant increase of the percentage of Ki-67-positive cells compared to the 3 day time point (Fig. 4G), signifying skin regeneration. A strong reduction of proliferative cells was noted at 98 days (Fig. 4G), being indicative of the post-regenerative phase of the skin reaction.

To determine how the loss of S-phase cells early after IR translates into skin degeneration, we determined the cellularity of the epidermis over time. The total number of keratinocytes along a 169 µm (unit) contour length of the epidermis in images of the biopsies (Fig. 4). Samples of control and the 4 h time point post IR displayed no significant variation of the number of keratinocytes per unit contour length (Fig. 4G), while a significant reduction in cellularity was noted from 3 to 49 days post IR relative to control (Fig. 4 B-D,H), reflecting IR-induced cell loss. At 98 days after irradiation, the numbers of keratinocytes per unit contour length had significantly increased relative to control (Fig. 4H), likely owing to the elevated proliferation after 28 days post IR (Fig. 4G) during compensatory hyperplasia.

#### Increased apoptosis in the irradiated epidermis

**Figure 5:**
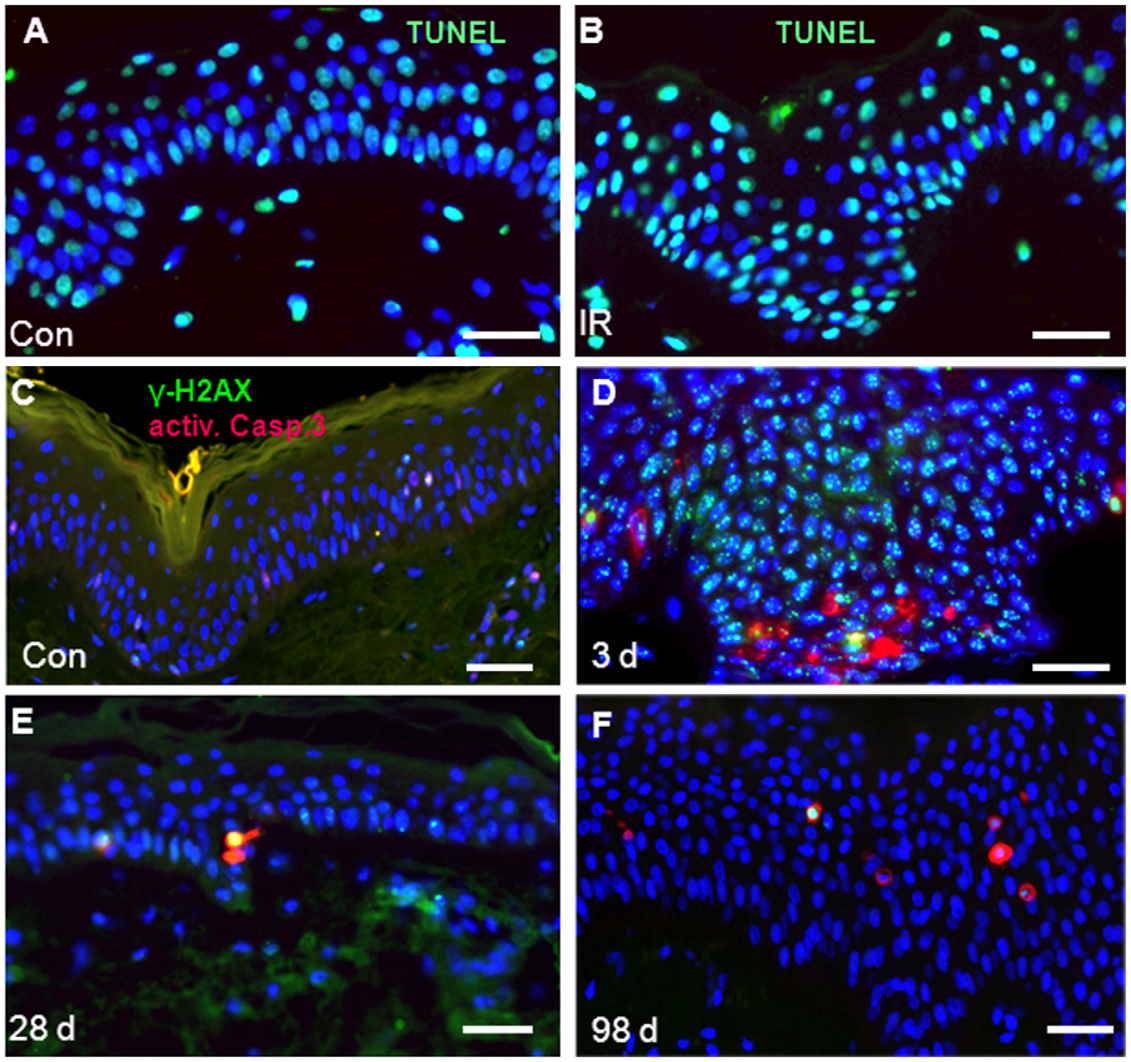
Acute IR exposure of the skin leads to caspase-dependent apoptosis. (A) TUNEL staining renders the epidermis largely positive for fragmented DNA in control (con) and (B) 50Gy-exposed skin sections (4 h post IR). (C-F) Immunofluorescence for activated-caspase 3 (a.casp.3; red) and γ-H2AX (green). Nuclei are counterstained with DAPI (blue). (C) Unirradiated control is largely lacking activated caspase 3-positive cells. (D) Three days post IR numerous activated-caspase 3-positive cells are present in the basal layer of the epidermis. (E) At 28 days post IR only few cells are positive for activated caspase 3. (F) 98 days post IR activated-caspase 3-positive cells often occur in the spinous layer of the epidermis (nuclei, blue) relative to the earlier time points. Activated-caspase 3-positive apoptotic cells at late time points often are also strongly positive for γ-H2AX nuclear staining (green; yellow due to signal overlap). Magnification bar: 20 µm.

To see whether and when cells undergo cell death after the high-dose exposure, we performed the TUNEL apoptosis assay that indicates DNA fragmentation in apoptotic cells [43], [44]. It was found that 40–60% of keratinocytes were positive for TUNEL signals in control and in irradiated skin samples (Fig. 5 A,B). We noted that epidermal cells generally showed a positive staining with the TUNEL assay, which agrees with previous studies [29], [45], [46] and reflects physiological DNA fragmentation in the course of keratinocyte cornification. Since the TUNEL assay proved not suitable to analyze IR-induced apoptosis, we next studied the apoptotic response by activated caspase-3 [47] immunofluorescence (Fig.5C-F). While we observed no difference in the frequency of activated caspase-3-positive cells in control samples and 4 hours post IR, there was a 17-fold increase of caspase-mediated apoptotic cells in the epidermal basal layer 3 days after IR (Fig. 5C,D), a highly significant increase relative to all other time points (Table 2). Twenty-eight days after exposure active caspase-3-positive cells were reduced again in number (Fig. 5 E) but their frequency was still more than 3-fold increased relative to control skin (Table 2), a difference that persisted up to 98 days after IR (Fig. 5F). These observations reveal that keratinocytes in the basal layer undergo increased caspase-dependent cell death early after acute high-dose irradiation, with apoptosis frequencies remaining significantly increased for months.

**Table 2:** Acute IR increases activated Caspase 3-dependent cell death.

time post IR	Con	4 h	3d	28d	49d	70d	98d
% of a.-casp.3-positive cells/169 µm unit area	0.21 (±0.19)	0.25 (±0.22)	**3.65** a (±1.70)	1.22 b (±0.63)	1.05 b (±0.80)	0.77 b (±0.49)	1.35 b (±0.34)

a Highly significant difference compared to all other time points (p<0.001).

b Significant difference to control and 4 h (p<0.05). Average and SD of 3 pigs and >300 nuclei from 12 sections/pig.

## **Discussion**

We studied the DNA damage response in Göttingen minipig pig skin after exposure to high dose and dose rate acute 50 Gy γ-irradiation [10] by γ-H2AX, 53BP1 and active ATM (Ser1981-P) immunofluorescence. Shortly after 50 Gy exposure all cells in the exposed skin region displayed DNA damage as indicated by massive H2AX phosphorylation and high numbers of radiation-induced 53PB1 foci. While the extensive γ-H2AX formation rendered RIF counting difficult or impossible in most nuclei 4 h post IR, a subset of nuclei still presented enumerable foci amounts, with γ-H2AX RIF numbers exceeding the average amount of 53BP1 foci, indicating the DNA damage response of 53BP1 binding may follow a different kinetics [34] as compared to kinase-dependent H2AX phosphorylation [48] after such high doses. Furthermore, the observed difference may relate to a limited nuclear pool of 53BP1 molecules that are able to associate with the chromatin surrounding DSBs shortly after IR exposure [35].

Human dermal cells exposed to 4 Gy IR displayed a rather uniform yield of residual 53BP1 foci levels after 24 h [14]. In this study we noted a similar 53BP1 RIF yield in the two 50Gy-irradiated pigs 4 and 20 h post IR, which contrasted with a more variable average γ-H2AX foci yield. Variations in γ-H2AX foci may result from individual genetic differences concerning the kinase and phosphatase components of the DDR [48]. Interestingly, the pig that received a 53 Gy surface dose still displayed higher average RIF numbers as compared to the two 50 Gy exposed animals, indicating that the DNA damage response is still able to address dose differences at this high dose range. This is in agreement with efficient DNA repair rates in mammalian cells exposed with up to several hundreds of Gy [49] and suggests that the DDR per se is not saturated at 50 Gy.

Twenty hours after *ex vivo* 50 Gy irradiation there was a regression of the diffusely γ-H2AX-positive chromatin to numerous distinct foci and the average RIF yield at 20 h post IR was exceeding the values of the 4 h enumerable cell fraction, indicating progression of the DDR. At 3 days post *in vivo* irradiation the average γ-H2AX and 53BP1 RIF numbers were further reduced and a subfraction of cells even lacked foci, signifying the progress of DNA repair in cornifying keratinocytes. Early after exposure there is a linear dose response regarding RIF numbers at doses up to 2 Gy [21], while this relationship is lost at higher doses leading to the accumulation of DSBs in single RIF [22]. At the more advanced time points after pig skin IR (28, 49, 70 days) average RIF numbers were largely reduced but still showed a more than 2-fold increase above the control values in all samples. Less than 1% of RIF-positive cells at >48 days post IR usually contained 1 or 2 DNA large DNA damage-related foci, whose size significantly exceeded that of RIF between 4 h and 28 days after IR, and that of replication-associated foci. In human skin the persistence of 53BP1 foci has been noted 24 h post 4 Gy IR [14] and up to 7 days in mouse tissues after 10 Gy acute photon IR [30]. Large persistent RIFs likely reflect the clustering of damaged chromatin regions [50], [51] that contain complex DNA damages involving base lesions, ssDNA and dsDNA breaks and abasic sites that are refractory to repair [27], [41], [52]. In agreement, we observed the continuous presence of active ATM-p kinase at these persistent RIFs, which indicates ongoing DNA damage signaling up to several months after IR. Persistent DNA damage-related foci have also been observed to accumulate as a consequence of cellular aging [36] or after high LET irradiation [53] and are considered as markers of lethal DNA damage (reviewed in [31]). Since corneocyte differentiation reflects a special type of programmed cell death [54], loss of cells carrying lethal DNA damage is expected to occur according to the cellular turnover of the epidermis, which in the minipig is about 47 days [8]. The observation that most of the persistent damage-related RIF disappeared about 70 days post IR may relate to the observation that lethal DNA damage can be carried over a few cell divisions [21], [55]. In this minipig model moist desquamation usually appeared after 70 days [11]. Hence, the associated cellular turnover may explain the control values of cells with DNA damage-related foci in skin samples taken at 96 days after IR.

In keratinocytes of the hair sheath, we noted a higher frequency of 53BP1 RIF-positive cells as compared to the epidermal keratinocytes at 70 days post IR. Data from mouse and pig suggest that the follicular epithelium is more radio-resistant than the epidermis [56]–[58], and the epidermal cells of the hair sheath may support radiation and conventional wound healing in pig skin by formation of dermal islands [6], [58]. Since dose built up will contribute to higher levels of DNA damage at greater depth, but higher RIF numbers in hair sheath cells were also seen near the surface of the skin, we favor a different DNA damage response in corneocytes and hair sheet cells that may relate to a longer epidermal turnover time in this compartment, a different rate of DNA repair, and/or a higher resistance of DNA-damage carrying hair sheath cells to apoptosis. This reasoning is corroborated by the observation that hair bulge cells display an increased rate of NHEJ repair and an increased resistance to DNA-damage-induced cell death [59]. Such features may support the persistence of hair sheath cells carrying lethally damaged chromatin.

Caspase-dependent cell death after high dose irradiation was 17-fold increased 3 days after exposure, largely among the mitotically active stem cells of the basal layer of the epidermis. At all subsequent time points up to 96 days we noted a >3-fold elevated rate of caspase-dependent apoptosis. Since caspase-mediated apoptosis was largely absent in control skin and is not activated during normal corneocyte formation [54], [60], the persistent IR-induced increase of cell death likely depleted the epidermal stem cell pool and induced the observed changes in epidermal cellularity and radiation wound formation. The fluctuating epidermal cell densities in combination with persistently elevated cell death likely underlie the degenerative, regenerative, and post-regenerative phases of the cutaneous radiation reaction [6], [58], [61].

In all, it is evident that investigation of persistent DNA damage-related RIF in the epidermis can provide information about a previous high-dose exposure and may thus be useful as an indicator of acute radiation exposure in radiological, nuclear and radiation accident scenarios.

## Materials and Methods

### Animals and Experimental Radiation Model

The experimental radiation Göttingen minipig model used in this study was used as described by Agay et al. [10]. For this study, formaldehyde-fixed skin biopsies were obtained within the radiation source-proximal 3.5×5 cm lumbar skin region “A” of Agay et al. (cf. Fig. 2 [10]) from 3 female Gottingen Minipigs (aged 14 to 16 months). The lumbar skin was exposed in two pigs to 50 Gy and one pig to 53 Gy gamma irradiation (Table 1) with a 60Co γ source (IRDI 4000; Alstom) at a dose rate of 0.6 Gy min-1 [10]. The dose delivered was controlled by an ionization chamber and several 1.5 cm×5 mm Al powder dosimeters (Desmarquest alumina [Al_2_O_3_], Desmarquest Fine Ceramics, France) fixed to the different regions traversed by the beam. In this investigation we only used biopsies of skin region “A” directly facing the beam and studied only the upper 0.9 mm. The doses noted will thus reflect the surface dose absorbed by the epidermal regions studied. Corrections for dose built up in different tissue depths (maximum expected in ∼5mm depth) were not made.

**Table 3:** Antibodies, dilutions and sources.

Antibodies	Dilutionused	Manufacturer
*Primary antibodies*		
Mouse anti-γ-H2AX, JBW301	1/500	Milipore
Rabbit anti-53BP1 NB100-304A3	1/300	Acris-antibodies
Monoclonal mouse anti-Ki67,clone MIB, M7240	1/100	Dako
Rabbit polyclonal anti-active Caspase 3,Ab13847	1/200	Abcam
Monoclonal mouse PhosphoDetectanti-ATM 2152-1	1/100	Calbiochem
Monoclonal mouse anti AE1/AE3cytokeratin	1/200	Dako
*Secondary antibodies*		
Goat anti-mouse Alexa 488	1/600	Dianova
Donkey anti-rabbit-Cy3	1/800	Dianova

Samples from three pigs were available for time points 4 hours, 28 days, 49, 70 and 98 days post irradiation. In two pigs we obtained each a sample of 50 Gy exposed and non-exposed skin 3 days after 50 Gy irradiation. Two 20 h samples were obtained by *ex vivo* irradiation of an explanted biopsy followed by 20h of organ culture in an incubator at 37°C and finally fixed over night in neutral buffered 4% formaldehyde, washed 3×5 min in PBS/0.1%Glycin, dehydrated, and embedded in Paraffin (Carl Roth) according to routine methods.

All animal trials were approved by the Animal Ethics Committee of the French Armed Forces Biomedical Research Institute (N°2008/24.0). All pigs were treated in compliance with the French legislation related to animal care and protection.

### Immunofluorescence and Image Analysis

Paraffin skin tissue sections (8 µm) of control and exposed skin biopsies were mounted on super-frosted slides and dried overnight at 37°C. Sections were dewaxed in xylene and hydrated in a graded series of alcohols. Slides were kept at 95°C for 40 min in 0.01 M sodium citrate (pH 5) and blocking was done in PBTG buffer [22]. The slides were incubated with the primary antibodies for 1 hour at 37°C in PBTG buffer [22], followed by 3×5 min washes in PBS and incubation with the secondary antibodies for 35 min. The antibodies, their sources and dilutions used are listed in table 3. After incubation with the secondary antibodies, sections were washed (3×5 min) in PBS at 37°C. Slides were supplied with 18ul Vectashield Mounting Medium (Vector labs) containing DAPI (4',6-diamidino-2-phenylindole) as DNA/nuclear counterstain and covered with a 24×60×mm cover slip.

Preparations were analyzed using a motorized Zeiss Axioplan 2 fluorescence microscope equipped with the ISIS fluorescence imaging system (MetaSystems, Altlussheim). Digital images of several optical planes of the sections were recorded and combined to a maximum projection images that were manually analyzed for the presence of foci/nucleus or for the presence of cells. The diameter of foci was digitally determined using the mean maximum contour radius function of the Metafer fluorescence imaging system (MetaSystems).

### Statistical Analysis

The results were analyzed using the t-test and the data were expressed as mean ± Standard deviation (SD) using graphpad software (graphpad.com). Skin samples of three mini-pigs per time point were analyzed; and three to 16 slides were scrod per mini-pig (i.e. 150–2000 cells/pig depending on cell density).

## Supporting Information

Figure S1 Replication-dependent DNA damage. Immunostaining for Ki67 (red) and 53BP1 (green) of keratinocytes (A–C) of the epidermis (basal layer is located at the lower end of the image) and (D) hair sheath. Nuclei are counterstained in blue. (A) Ki67-positive cells residing at the basal layer of non-irradiated S-phase keratinocytes show very small 53BP1 foci (arrow; inset) indicative of replication-dependent DNA damage. (B) Twenty-eight and (C) 70 days after IR S-phase cells are found among the basal cells of the epidermis (arrows). (C) Previous irradiation is indicated by the large persistent foci in nuclei of epidermal keratinocytes and (D) hair sheet epithelial cells even 70 days after IR. Magnification bar: 20 µm.
(TIF)

Figure S2 Detection of keratinocytes in skin punch biopsies. Immunostaining for cytokeratin (green) specifically labels keratinocytes in (A) the superficial epidermis and (B) of the epithelial hair sheath cells 28 days post irradiation.
(TIF)
