# Supplementary figures and images for: Persistent DNA Damage after High Dose In Vivo Gamma Exposure of Minipig Skin
Source: PLoS One. 2012 Jun 27;7(6):e39521. doi: 10.1371/journal.pone.0039521 (PMC3384646; doi:10.1371/journal.pone.0039521)

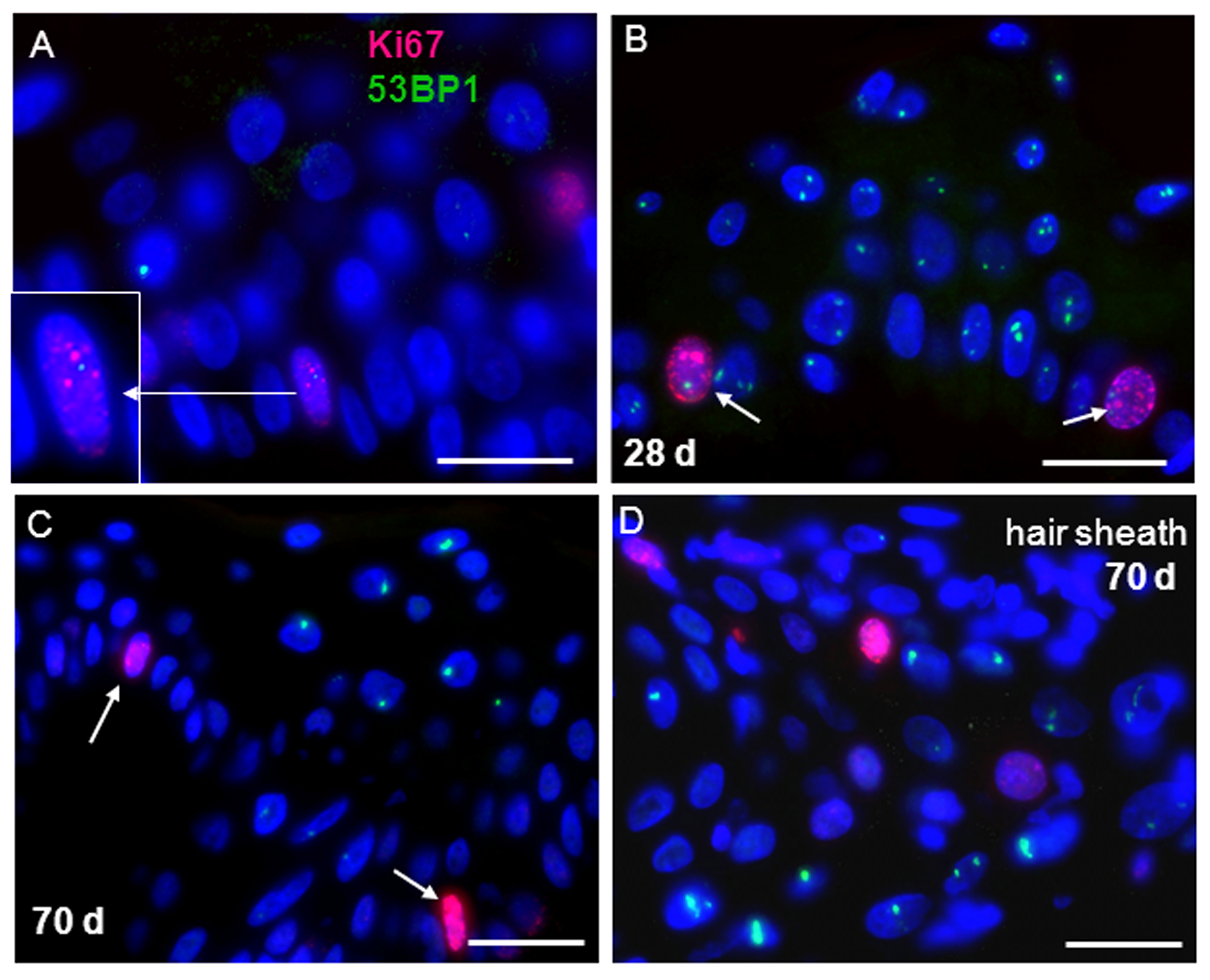

Supplement: Figure S1 — Replication-dependent DNA damage. Immunostaining for Ki67 (red) and 53BP1 (green) of keratinocytes (A–C) of the epidermis (basal layer is located at the lower end of the image) and (D) hair sheath. Nuclei are counterstained in blue. (A) Ki67-positive cells residing at the basal layer of non-irradiated S-phase keratinocytes show very small 53BP1 foci (arrow; inset) indicative of replication-dependent DNA damage. (B) Twenty-eight and (C) 70 days after IR S-phase cells are found among the basal cells of the epidermis (arrows). (C) Previous irradiation is indicated by the large persistent foci in nuclei of epidermal keratinocytes and (D) hair sheet epithelial cells even 70 days after IR. Magnification bar: 20 µm. (TIF) [file pone.0039521.s001.tif]

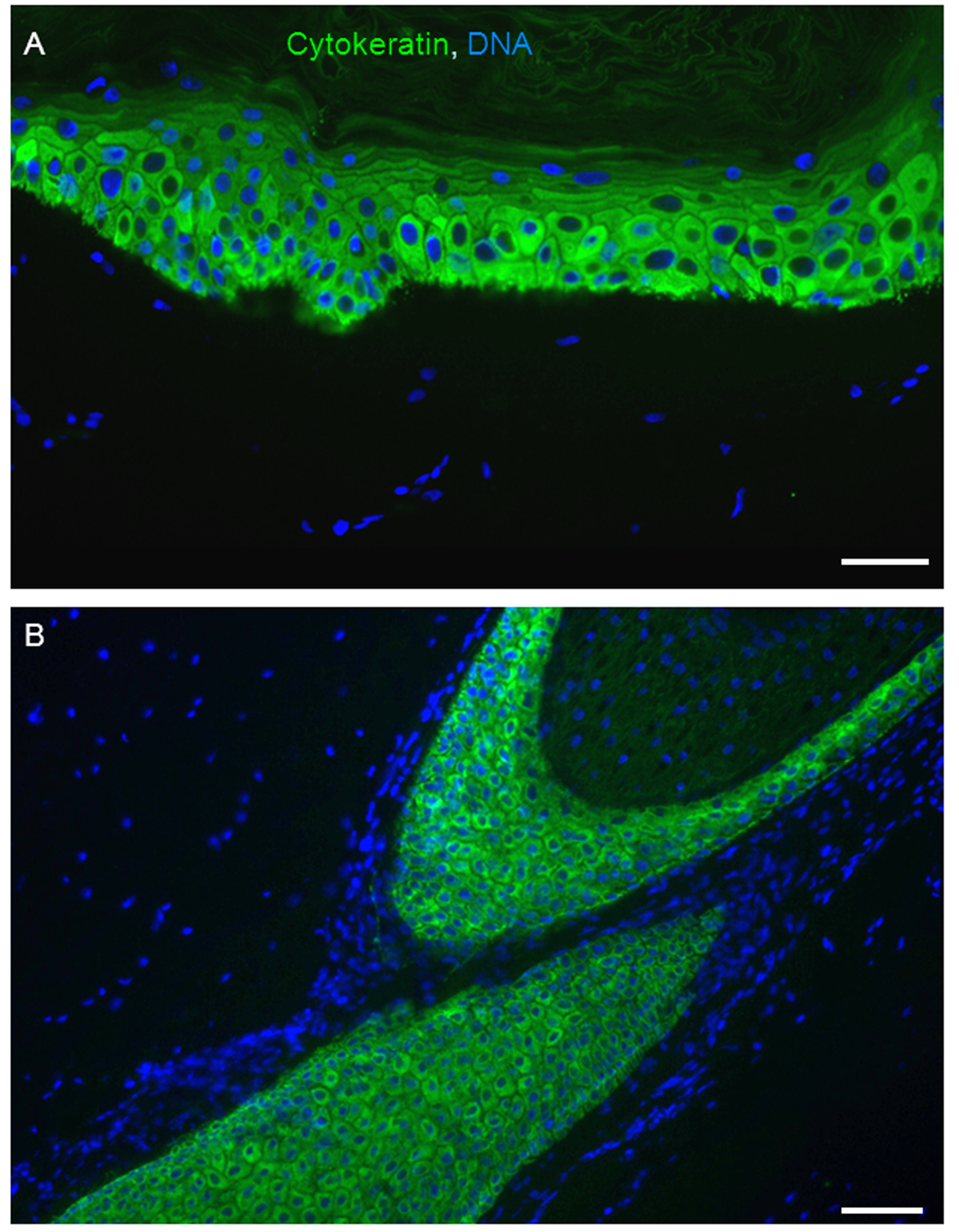

Supplement: Figure S2 — Detection of keratinocytes in skin punch biopsies. Immunostaining for cytokeratin (green) specifically labels keratinocytes in (A) the superficial epidermis and (B) of the epithelial hair sheath cells 28 days post irradiation. (TIF) [file pone.0039521.s002.tif]
